# Factors associated with seeking preventive dental care: an integrative model exploration of behaviors in Mexican immigrants in Midwest America

**DOI:** 10.1186/s12903-018-0502-x

**Published:** 2018-03-12

**Authors:** Jonathan T. Macy, Elizabeth A. S. Moser, Adam T. Hirsh, Patrick O. Monahan, George J. Eckert, Gerardo Maupomé

**Affiliations:** 1 Department of Applied Health Science, Indiana University School of Public Health, 1025 East 7th Street, Room 116, Bloomington, IN 47405 USA; 2 Indiana University School of Medicine, Indianapolis, IN USA; 3 Indiana University-Purdue University, Indianapolis, IN USA; 4 Indiana University Richard M. Fairbanks School of Public Health, Indianapolis, IN USA; 5 Indiana University Network Science Institute, Bloomington, IN USA

**Keywords:** Mexican-American, Integrative model of behavioral prediction, Health behavior, Oral health, Barriers, Preventive care

## Abstract

**Background:**

Mexican immigrants in the United States suffer from poor oral health. The objective of the current study was to explore the utility of applying theory-based factors associated with seeking preventive dental care in a sample of Mexican American adults.

**Methods:**

Data were collected from a cross-sectional survey of a sample of 157 people of Mexican origin (64% female; age 34 ± 11 years) recruited primarily from church congregations and lay community organizations in Central Indiana. Using the Integrative Model of Behavioral Prediction as the guiding framework, structural equation modeling was used to test factors associated with intention to seek preventive dental care.

**Results:**

Attitude towards seeking preventive dental care (estimate = 0.37; *p* < .0001) and self-efficacy for seeking preventive dental care (estimate = 0.68; p < .0001) were associated with intention to seek preventive dental care. The association between dental beliefs and intention to seek preventive dental care was mediated by attitude and self-efficacy (indirect effect = 0.26, *p* = .002), and the association between past behavior and intention to seek preventive dental care was mediated by self-efficacy (indirect effect = 0.26, *p* = .003).

**Conclusions:**

These findings suggest that interventions to increase preventive dental care seeking behavior among Mexican Americans should focus on changing attitudes toward seeking preventive dental care and on increasing self-efficacy to seek preventive dental care. Findings also support the use of interventions to influence dental beliefs.

## Background

Multiple reasons contribute to the complex scenario of oral health disparities affecting people of Mexican origin in the United States (US) (generically called Mexican-Americans (MAs) for simplicity in this manuscript). With limited access to care, high-risk diet and lifestyle, and low ability to navigate health care systems in the list of likely reasons, oral health behaviors may be one major component in the MA oral health disparities scenario [1–6]. Although positive and detrimental behaviors coexist to various degrees in MAs, they often fail to engage in behaviors that promote good oral health, and they appear to over-engage in behaviors that are harmful [5, 6]. Compared to the general population, Hispanics have less access to health care, lower educational attainment, often lack health insurance, are more likely to live in poverty, and face cultural barriers that hinder their ability to navigate health care systems [7, 8]. These challenges place them at higher risk for oral health problems. MAs have often been reported to have worse oral health and the lowest use of dental care services compared to other Hispanic children and adolescents [9–11]. The trends for adult MAs are similar: they are not likely to receive dental care in a timely manner and have a high percentage of unmet dental needs [10]. More recent descriptions of periodontal and dental health among Latinos suggested, however, that substantial variation exists across nationality of origin, age, and sex [12, 13]. An important question in this context is how to tease out the components of a complex scenario in which personal decisions take place in a multifaceted environment influencing how people address health issues.

**Fig. 1:**
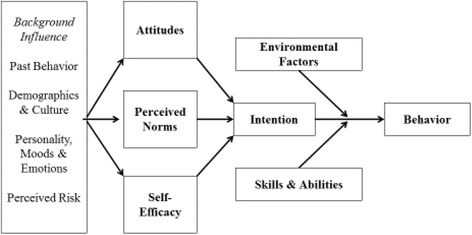
Integrative Model of Behavioral Prediction Framework

Given that the MA population makes up about two-thirds of Latinos in the US [14], and the documented disparities in dental care utilization and outcomes [5, 6], it is of public health significance to better understand factors affecting disparities. Such barriers to dental care likely include economic, socio-cultural, structural, and personal factors. We propose that to better understand the barriers faced by MAs, the factors involved should be explicitly assembled under the umbrella of a health behavior model that facilitates conceptual characterization and practical measurement of the relevant attitudes, behaviors, and norms. To do that, the current research was placed under an Integrative Model of Behavioral Prediction (IM) framework (Fig. 1) [15, 16] to evaluate the factors that are associated with MA consumers seeking periodic preventive care in the dental office. According to the IM, intention, the perceived likelihood of engaging in a particular behavior, is the most important predictor of that behavior. Intention is determined by a combination of attitude towards engaging in the behavior, perceived normative pressure to engage in the behavior, and self-efficacy to engage in the behavior. Further, it is postulated that attitudes, perceived norms, and self-efficacy mediate the relationships between background variables and intention [17]. Background variables represent more upstream factors, such as demographic, cultural, personality, or other individual differences, that may influence intention through attitudes, perceived norms, and self-efficacy. A large body of research demonstrates that the Theory of Reasoned Action (TRA) and Theory of Planned Behavior (TPB) – theoretical precursors to the IM – predict a range of health behaviors and intentions to engage in health behaviors, including substance use, contraceptive use, exercise, sexually transmitted illness prevention, cancer screening, and nutritional choices [18]. Although this research has been criticized, particularly for its reliance on correlational designs, many studies have utilized this theoretical approach to identify potential targets for interventions to encourage preventive health behaviors [19–22]. Indeed, several intervention trials indicate that changing TRA/TPB/IM constructs yields subsequent changes in behaviors [23–30]. However, to our knowledge, the IM framework has not been extensively applied to oral health themes.

Having periodic preventive care delivered in the dental office is often cited as the hallmark of early identification of disease, timely implementation of secondary prevention measures, and appropriate management of risk to oral diseases [31]. Moreover, adherence to a consistent schedule (ideally, at least annually) is often associated with positive oral health outcomes. We have established separately that MAs’ adherence to seeking preventive dental care in dental offices is often inadequate. This is because preventive dental care is often a haphazard occurrence, undertaken merely as an addition when episodic attendance takes place because of acute problems or in the close aftermath of addressing symptomatic clinical presentations [5, 6]. The cost of periodic preventive dental care is often seen as a luxury because no ‘real’ dental issue needs to be fixed [5, 6]. Building on these previous findings, the objectives of the present exploratory research were to test theory-based predictors of intention to seek preventive dental care and to test whether the relationships between background variables and intention to seek preventive dental care were mediated through attitudes, perceived norms, or self-efficacy in a sample of MAs in Central Indiana.

## Methods

### Participants and procedure

We recruited MA adults living in an urban environment through advertising at health fairs, churches and religious gatherings, the waiting room in the Mexican Consulate in Indianapolis, and community functions in 2014. Written informed consent from participants was obtained. Participants responded to the survey in English or Spanish, according to their preference, in sessions that lasted an average of 30–45 min. Participants received US $30 compensation.

Of the 191 people approached who met the inclusion/exclusion criteria, (160) 84% agreed to participate. Those who did not agree to participate were usually pressed for time when attempted to be recruited. Inclusion criteria were 21 years of age or older, immigrant status with more than three years living in the US, and able to read and write either English or Spanish. Exclusion criteria were mental or physical conditions that impaired participation in the study. To assess the exclusion criteria, we asked potential participants if they were unable to read or hear sufficiently well to maintain a conversation, listen to instructions, or read a document.

### Measures

Intention to seek preventive dental care was a latent variable composed of two items (“I plan to go regularly to a dentist for preventive care,” measured on a five-point scale from strongly disagree to strongly agree, and “I am likely to go regularly to a dentist for preventive care,” measured on a five-point scale from highly unlikely to highly likely). Attitude towards seeking preventive dental care was a latent variable composed of six items measured on five-point scales that assessed going to a dentist regularly for preventive care as good versus bad, harmful versus beneficial, unpleasant versus pleasant, foolish versus wise, unnecessary versus necessary, and a poor investment versus a good investment. Perceived norms regarding seeking preventive dental care was a latent variable composed of three indicators. The first two indicators were items measured on five-point scales from strongly disagree to strongly agree (e.g., “People who are important to me think I should go regularly for preventive care”). The third indicator was computed by taking the mean of the available responses to four items measured on five-point scales (“My friends probably think I should go regularly to the dentist for preventive care”, “My family probably thinks I should go regularly to the dentist for preventive care”, “My spouse probably thinks I should go regularly to the dentist for preventive care”, and “My boyfriend or girlfriend probably thinks I should go regularly to the dentist for preventive care”).This procedure was followed because of missing data on these items, but each participant responded to at least one of these four items. Self-efficacy to seek preventive dental care was a latent variable composed of three items measured on five-point scales from strong disagree to strong agree (“I am confident I could go regularly to a dentist for preventive care,” “Whether I go regularly to a dentist for preventive care is entirely under my control,” and “I feel I know how to go regularly to a dentist for preventive care.”). Dental beliefs was a latent variable composed of three items measured on five-point scales from strongly disagree to strongly agree (extent of agreement with statements that sooner or later everyone will need urgent dental treatment, it is a fact of life; that as I get older, I expect I will lose some of my teeth; and that most children will eventually have caries/cavities). Finally, participants reported whether they had dental insurance and when they had last visited a dental office. For analyses, a binary variable was created for visited a dental office within the last year versus all other responses.

### Data analysis

Guided by the IM framework, structural equation modeling was used to test attitudes, perceived norms, and self-efficacy as predictors of intention to seek preventive dental care. In addition, demographics, having dental insurance, past behavior, and dental beliefs were tested as background variables that were hypothesized to influence intention to seek preventive dental care through attitudes, perceived norms, and self-efficacy. Analyses were conducted using SAS software, Version 9.4 for Windows (© SAS Institute Inc., Cary, North Carolina, US).

## Results

Of the 160 participants recruited, 3 were excluded from analyses due to missing data on one or more of the background variables included in the structural equation modeling analysis. This resulted in a final sample size of 157 (64% female; mean age = 34.4, standard deviation = 11.1).

**Fig. 2:**
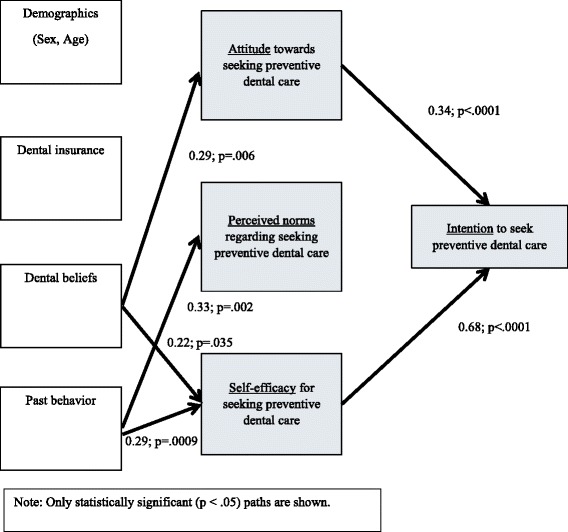
Path coefficients for model with background variables as precursors to the integrated model of behavioral prediction analysis of intention to seek preventive dental care among Mexican-American adults in Indiana, 2014 (*N* = 157)

Figure 2 displays the results for the structural equation modeling analysis. In terms of the background variables tested, dental beliefs were associated with attitude and self-efficacy, and past behavior was associated with perceived norms and self-efficacy. In terms of the three global IM constructs (attitudes, perceived norms, and self-efficacy), attitude and self-efficacy were associated with intention to seek preventive dental care. We also tested the theory-based indirect relationships between the background variables and intention to seek preventive dental care. We found that the association between dental beliefs and intention to seek preventive dental care was mediated by attitude and self-efficacy (indirect effect = 0.26, *p* = .001), and the association between past behavior and intention to seek preventive dental care was mediated by self-efficacy (indirect effect = 0.28, *p* = .0001). Finally, we estimated another model that included the direct effects of sex, age, dental insurance, dental beliefs, and past behavior on intention. All of the statistically significant paths shown in Fig. 2 were replicated. There were no statistically significant direct effects of the background variables on intention to seek preventive dental care.

## Discussion

The first finding of note was that, of the three global IM constructs (attitudes, perceived norms, and self-efficacy), attitude towards seeking preventive dental care and self-efficacy to seek preventive dental care were related to intention to seek preventive dental care in this sample of MAs. Perceived norms regarding seeking preventive dental care, however, was not associated with intention. These findings are consistent with earlier studies [32, 33] that have found that the TPB (the precursor to the IM) works well as a conceptual framework for understanding oral health behaviors.

The current study’s results have implications for the design of interventions to increase preventive dental care seeking behaviors among MAs. Specifically, interventions should focus on changing attitudes toward seeking preventive dental care and on enhancing self-efficacy to seek preventive dental care. While there is no unequivocal evidence supporting the effect of preventive visits on improved oral health outcomes, consistent and periodic preventive dental visits remain a centerpiece of professional recommendations [31]. Indeed, diagnostic and preventive procedures account for more than 75% of all dental care services received [34], and such a trend is likely to remain stable [35]. Questions about the evidence base for professional recommendations regarding seeking periodic dental care are beyond the scope of the present manuscript.

The current study also examined the role of background variables on intention to seek preventive dental care. Based on the IM, we expected the background variables to influence intention through attitudes, perceived norms, or self-efficacy. Two of the background variables tested had statistically significant indirect effects on intention. First, the associations between dental beliefs and intention were mediated by attitude and self-efficacy. That is, our findings suggest that MAs’ dental beliefs are related to their attitude and to their self-efficacy about seeking preventive dental care. Therefore, messages that target dental beliefs of MAs could lead to meaningful changes in their intention to access preventive dental services through either an attitudinal pathway or by increasing self-efficacy. Second, the association between past behavior and intention was mediated by self-efficacy. Participants who had visited a dental office in the past year had higher self-efficacy, which in turn was related to intention to seek preventive dental care.

This study has some limitations that should be considered. First, the sample size is relatively small. However, for an exploratory study, the sample size was adequate to identify statistically significant theory-based relationships among constructs. This represents an important first step in applying the IM framework to understanding oral health behaviors among MAs. Second, the sample is limited to MAs living in urban communities in Central Indiana who were recruited largely from Catholic parishes or other community-oriented groups. Thus, generalizing results beyond the current exploration of the IM framework to ascertain certain components of seeking preventive dental care is unwarranted. Third, our analyses are primarily based at the individual level and do not include important structural influences such as public policies and health care system factors. Fourth, the cross-sectional non-experimental design precludes solid suggestions about causality, and therefore possible bi-directional relationships must be acknowledged. For example, it is possible that higher (unmeasured) pre-visit self-efficacy influenced a completed visit to the dental office, post-visit self-efficacy, and intention to seek care in the future. Moreover, we acknowledge that because of the cross-sectional nature of the data, the background variables, theoretical mediators, and intention to seek preventive dental care were all measured at the same time. Future research with large, representative, longitudinal samples that include system-level as well as individual-level data is clearly warranted to overcome these limitations.

This study is also characterized by multiple strengths. First, there are few studies in the new, evolving immigration entry point in the American Midwest; this has become a favored gateway for Hispanic immigrants, as opposed to other locations in which the Hispanic population has traditionally taken residence. As a consequence, the Midwest has recently experienced much faster growth than other Hispanic areas in the US [36, 37]. Second, the novel use of the IM framework aids in our ability to disaggregate factors underlying oral health disparities. Third, the sample is comprised entirely of MAs thus controlling for nationality of origin and avoiding aggregating all Hispanics in one large category, which has unknown effects. Finally, our study yields insights into clinical care implications and supports a culturally sensitive understanding of oral health.

## Conclusions

These findings suggest that interventions to increase preventive dental care seeking behavior among MAs should focus on changing attitudes toward seeking preventive dental care and increasing self-efficacy to seek preventive dental care. Findings also support the use of interventions to influence dental beliefs.

## Abbreviations

IM: Integrative Model of Behavioral Prediction
MA: Mexican American
TPB: Theory of Planned Behavior
TRA: Theory of Reasoned Action
US: United States
